# The Safety INdEx of Prehospital On Scene Triage (SINEPOST) study: the development and validation of a risk prediction model to support ambulance clinical transport decisions on-scene—a protocol

**DOI:** 10.1186/s41512-021-00108-4

**Published:** 2021-11-08

**Authors:** Jamie Miles, Richard Jacques, Janette Turner, Suzanne Mason

**Affiliations:** 1 CURE Group, School of Health and Related Research, University of Sheffield, Regent Court, 30 Regent Street, Sheffield, S1 4DA UK; 2 Yorkshire Ambulance Service, Brindley Way, Wakefield, WF2 0XQ UK; 3 School of Health and Related Research, University of Sheffield, Regent Court, 30 Regent Street, Sheffield, S1 4DA UK

**Keywords:** Ambulance, EMS, Emergency, Triage, Acuity, Machine learning, Logistic regression, XGBoost

## Abstract

**Background:**

Demand for both the ambulance service and the emergency department (ED) is rising every year and when this demand is excessive in both systems, ambulance crews queue at the ED waiting to hand patients over. Some transported ambulance patients are ‘low-acuity’ and do not require the treatment of the ED. However, paramedics can find it challenging to identify these patients accurately. Decision support tools have been developed using expert opinion to help identify these low acuity patients but have failed to show a benefit beyond regular decision-making. Predictive algorithms may be able to build accurate models, which can be used in the field to support the decision not to take a low-acuity patient to an ED.

**Methods and analysis:**

All patients in Yorkshire who were transported to the ED by ambulance between July 2019 and February 2020 will be included. Ambulance electronic patient care record (ePCR) clinical data will be used as candidate predictors for the model. These will then be linked to the corresponding ED record, which holds the outcome of a ‘non-urgent attendance’. The estimated sample size is 52,958, with 4767 events and an EPP of 7.48. An XGBoost algorithm will be used for model development. Initially, a model will be derived using all the data and the apparent performance will be assessed. Then internal-external validation will use non-random nested cross-validation (CV) with test sets held out for each ED (spatial validation). After all models are created, a random-effects meta-analysis will be undertaken. This will pool performance measures such as goodness of fit, discrimination and calibration. It will also generate a prediction interval and measure heterogeneity between clusters. The performance of the full model will be updated with the pooled results.

**Discussion:**

Creating a risk prediction model in this area will lead to further development of a clinical decision support tool that ensures every ambulance patient can get to the right place of care, first time. If this study is successful, it could help paramedics evaluate the benefit of transporting a patient to the ED before they leave the scene. It could also reduce congestion in the urgent and emergency care system.

**Trial Registration:**

This study was retrospectively registered with the ISRCTN: 12121281

**Supplementary Information:**

The online version contains supplementary material available at 10.1186/s41512-021-00108-4.

## Background

Demand in the emergency care system is increasing. In prehospital care, this translates to an increase of around 5% per annum and in the emergency department (ED) is around 3–6% [1, 2]. When the ED is busy, ambulance crews can be held in a queue at the ED and this is known as offload delay. In the winter of 2019/2020 in England, there were 137,009 offload delays of between 30 and 60 min and 39,304 delays of over an hour [3]. With crews held at the ED, it reduces the prehospital fleet capacity to respond to emergencies and subsequently puts patients in the community at risk.

One of the contributors to demand in the system is the case-mix of patients that access emergency care. The majority of ambulance service patients require fewer critical interventions and more community-based care [4, 5].

This appears to be at a juxtaposition to the training of paramedics. Numerous studies have found that there is role confusion when paramedics are presented with a low-acuity patient, as their foundational knowledge and education was rooted in emergency care [6–11]. This meant that decisions to leave a patient at home (non-conveyance) are the most complex to make and this was further compounded by a perceived lack of managerial support [6].

As a result, transport decisions are not always accurate and there could be between 9 and 32% avoidable conveyances to the ED [4, 12–14]. Miles et al. used vignettes of real patient journeys and asked paramedics to make transport decisions. They found that there was clear agreement between the sample paramedics (*k*=0.63), and the overall accuracy in decision-making was 0.69 (95% confidence interval (CI) 0.66–0.73). Reassuringly, the sensitivity for transport decisions was high (0.89, 95% CI 0.86–0.92) meaning that there were few decisions not to convey a true emergency. However, the specificity was 0.51 (95% CI 0.46–0.56) meaning that almost half of the sample decided to transport a low-acuity patient [15].

There is a paucity of evidence for transport decision-support tools for paramedics. One example, which has been adopted by numerous ambulance services, is the paramedic pathfinder tool [16, 17]. This was developed using a Delphi approach with a multidisciplinary team of experts. The tool was user tested in 2014 on a sample of 481 patients (361 medical patients and 114 trauma). Results for medical patients showed a sensitivity of 0.94 (95% CI 0.91–0.97) and specificity of 0.58 (95% CI 0.49–0.66). For trauma sensitivity was 0.96 (95% CI 0.88–0.99) and specificity 0.6 (95% CI 0.48–0.72). These results are not a significant improvement on paramedics making their own decisions, which limits the usefulness of the pathfinder tool.

A recent systematic review by Miles et al. looked at whether computer algorithms could triage the acuity of all patients entering emergency care and support decision making [18]. They found 92 models from 25 studies. The review demonstrated that it is possible to triage patients accurately using machine-learning algorithms but only six studies had a prehospital focus. Two studies demonstrated that prehospital variables could predict hospital admission. Meisel et al. used logistic regression to create an admission prediction score with a C-statistic of 0.80 [19]. Li and Handly used a panel of algorithms, with the most successful being a modified support vector machine, which had an accuracy of 0.81 [20].

Seymour et al. used logistic regression to derive a risk score to predict critical illness in prehospital patients. Their model had a C-statistic of 0.77 (95% CI 0.76–0.78) [21]. van Rein developed a triage model for trauma patients and found that the model had a C-statistic of 0.82 (95% CI 0.81–0.83) [22].

These studies have demonstrated that it is possible to develop accurate models prehospital for triaging patients using clinical data. However, they have been developed to predict high-acuity patients as opposed to low-acuity.

### Objectives

#### Primary research question

Can ambulance service clinical data predict an avoidable attendance at the ED in adults?

#### Primary objective

To build risk prediction models using prehospital clinical data as input candidate variables, and ED experience as the output variable.

#### Primary outcome measure

An avoidable attendance at ED as defined by O’Keeffe et al. (2018). This is described as ‘First attendance with some recorded treatments or investigations, all of which may have reasonably been provided in a non-emergency care setting, followed by discharge home or to GP care’ [13].

#### Secondary research questions

What is the simulated transportability of the model derived from the primary outcome?

#### Secondary objectives

Evaluate model test performance under different spatial test sets.

Compare the different models for accuracy and feasibility to embed in practice.

#### Secondary outcome measure

There are no secondary outcome measures

## Methods and analysis plan

This protocol has used the Transparent Reporting of a multivariable prediction model for Individual Prognosis or Diagnosis (TRIPOD) guidelines in its structure [23]. The final study publication will also adhere to these guidelines.

### Source of data

This study is an observational cohort study using retrospective data. All patients attended by Yorkshire Ambulance Service (YAS) have an electronic patient care record (ePCR) completed by the paramedic treating them. This contains all demographic and clinical data relating to that episode. If the patient is transported to an emergency department (ED), a similar record is created for their ED episode containing all demographic and clinical information. These two records will be linked together to create a single patient journey for each patient from the moment the paramedic arrived on scene, to their outcome at ED. This cohort is the primary analysis cohort and will be used for model development, and internal-external validation.

The data collection period started on 1st July 2019, as this was the earliest date that Yorkshire Ambulance service had a region-wide rollout of the electronic patient care record (ePCR). The end date was the 29th February 2020. The end date was chosen for a maximum sample size, without the data being compromised by the COVID-19 pandemic. The data was not extracted until after the end date.

### Participants

The study is set in pre-hospital care but uses ED experience as the outcome. There is one ambulance service involved (YAS) and sixteen EDs throughout Yorkshire.

Patients were eligible for inclusion if they were over 18 years old at the time of attendance and had a completed record in the ambulance service data, and the ED data (if they were transported). The patients can be described as largely ‘unselected’. This means all patients are eligible, irrespective of any demographic or disease process. The only restriction in selection is age being over eighteen. This is due to ambulance service policies often mandating the transport of children to hospital.

### Outcome

The outcome of the model is a non-urgent attendance at the ED. The reference standard is described by O’Keeffe et al. who state: “first attendance with some recorded treatments or investigations all of which may have reasonably been provided in a non-emergency care setting, followed by discharge home or to GP care.” [13]. This definition has been transformed into a data-driven coded definition and is found in the routinely collected Emergency Care Data Set (ECDS), and the former Commissioning Data Set (CDS) in the UK [24]. The full coded definition can be found in the supplementary material. The definition is calculated by examining each patient’s ED experience across six variables. These are department type, attendance category, arrival mode, investigations, treatments and discharge status. For a patient to be coded as non-urgent, they need to only have experienced the values recorded in the definition. As an illustrative example, please see Table 1.

**Table 1:** Illustrative example of how the definition is applied to patients

Variable	Patient 1	Patient 2	Patient 3
Department type	Type 1	Type 1	Type 1
Arrival mode	Ambulance	Ambulance	Ambulance
Attendance category	First	First	First
Investigations	None	Urinalysis, pregnancy test	Urinalysis, chest X-ray
Treatments	Guidance/advice	Recording vital signs, prescription	None
Discharge status	Discharged	Discharged	Discharged
Non-urgent attendance	Yes	Yes	No

The justification for this reference standard is that it has been adopted by National Health Service (NHS) Digital as the accepted definition of non-urgent attendance at the ED. There are two modifications to this standard for this study in that arrival mode was defined as non-ambulance arrival, but this has been changed to ambulance arrival only. The included investigations and treatments have been expanded to reflect the practice of the ambulance service and the provision of primary care. The modifications were decided by an expert group.

### Candidate predictors

In order to inform the protocol and the sample size calculation, a combination of previously published literature and an exploration of prehospital data was used (not used in model development). Previous prediction modelling studies of emergency triage have published variables that were significant in their models. Physiological variables for example pulse rate and blood pressure appear to be the most significant predictors of acuity. This is followed by patient comorbidities and whether the case originated from a non-residential setting [25, 26]. A sample of ambulance ePCRs (114,715) was used to identify clinically useful candidate variables in the ambulance data. The model is designed to be pragmatic so if a candidate predictor had more than 30% missing data it was removed. If a variable was likely to contain missing data as it did not occur (judged by evidence of a positive class within the variable) then ‘none’ was imputed. For example, the variable ‘drug_name’ only gets completed if a drug is given. In the sample data, there were 106,052 (93%) missing values in this field, and in rest a specific drug was named (e.g. Adrenaline 1:1000). Therefore, ‘no drug’ can be imputed into the missing values as it is assumed nothing was administered. This is the same process that NHS Digital use in their definition of the outcome. In the sample, there were 503 variables in the data. Four-hundred and forty-three variables had more than 30% missing data and were excluded from the analysis. This left 60 variables available for analysis, physiological variables, interventions, treatments and source of call (residential home, care home etc.) were all included.

### Statistical analysis methods

An XGBoost algorithm will be used to develop the models. This has been chosen as it can accept missing data in the candidate variables during model development, which may have an advantage when transforming it into an electronic decision support tool. Another strength of a gradient boosted algorithm is that it can increase the cost of errors on a minority class being predicted, which is a benefit in a dataset with a class imbalance. It also has a strength over neural networks when handling tabular data, which is how the data will be structured in the analysis, and finally, it is fast at processing data compared with other machine learning algorithms. This is important when it comes to the number of models required in a grid search (discussed later).

### Sample size

Minimum sample size was derived using ‘*pmsampsize v1.1.0*’ package for R v3.6.1 for Windows [27]. This package is based on the work of Riley et al. for calculating sample sizes for prediction models [28, 29]. A systematic review of similar outcomes including discharge from ED, critical care requirement and hospitalisation informed the sample size [18]. From these studies, the average C-statistic was 0.80. Candidate variables were examined in the non-conveyed data to estimate parameters. A limitation with XGBoost is the handling of categorical variables. This requires each category within a variable to become its own binary variable which has a single degree of freedom. There was a total of 637 parameters identified in the data. The total parameters per variable can be found in the supplementary material. A study examining avoidable conveyances reported a conservative estimate of 9% avoidable conveyances in the same population as this study [13]. The C-statistic was transformed into a Cox-Snell R^2^ via the *pmsampsize* package [30]. The arguments used in *pmsampsize* were therefore type = binary, C-statistic = 0.80, parameters = 637 and prevalence = 0.09. This gave an estimated minimum sample size of 52,958, with an anticipated 4767 events and an EPP of 7.48. A frequency analysis of the actual ePCR dataset shows there were 328,763 patients eligible for inclusion. However, the outcome measure requires data linkage, with unsuccessful linkage causing cases to be excluded [31]. This will likely result in fewer incidents to be included in the study.

### Missing data

Missing values within the candidate variables will be handled as described above. If a variable contains missing values, it will be assessed as to whether they are the negative class within the variable as opposed to missing. This will be done by analysing the variable in the context of the ePCR to check if the field is only completed if the event happened. If this is the case, the missing values will be imputed with ‘none’. Once this has been completed, any variable with more than 30% missing data will be excluded from analysis, as this provides evidence that the variable is not routinely collected and could cause model failure in practice, if included. Once the candidate predictors have been assessed for missing values, missing fields in each case will be examined. If any case does not have the outcome variable, but an ED record present, they will be excluded from the analysis. During model development, missing data will be handled via sparsity-aware split finding. This happens as part of the XGBoost algorithm. It uses non-missing data at each split to generate a default split. Then if there is missing information at the node, the algorithm defaults down the branch [32].

### Variable handling

Nominal, ordinal and binary will be treated in the same way and will be one-hot encoded into binary variables. Continuous variables will remain in their natural format. Feature engineering of a previous attendance within 24 h of the current incident will also be engineered into a binary variable. The rationale to create this variable is so the model is aware of a second contact with the emergency service (ensuring it accounts for repeat presentations, which can indicate a missed problem the first time). All variables will be included in the model development initially. Then, the model will undergo Recursive Feature Elimination (RFE). A feature importance score will be assigned to each feature and the least important stripped from the model. The model will be developed again with the same default hyperparameters but with one less feature. This repeats with the accuracy being recorded each time. The optimum set of features to take forward into model development will be identified by the model with the highest C-statistic with the default parameters. The data will be subset to only the features that yielded the optimum C-statistic and this subset will be used for all further modelling.

### Hyperparameters

To prevent model overfitting, there will be tuning of hyperparameters before developing each model. This will be done with a fixed set of values for certain hyperparameters within a restricted search space. In order to optimise the search space for the grid search, individual hyperparameters will be tuned on the whole dataset sequentially and the 3 best performing values within each hyperparameter will be taken forward to create the restricted grid search space for all subsequent modelling. The following hyperparameters will be tuned:

To control model complexity the following hyperparameters will be tuned:

*max_depth*—The maximum depth of each tree. The initial search space will be between 2 and 10, with intervals of 1.

*min_child_weight*—This is a threshold for whether to continue partitioning a tree based on the sum of instance weight, with larger numbers creating a more conservative model. The initial search space will be 1 and 10, with intervals of 1.

*gamma*—Also known as *min_split_loss*. Like *min_child_weight*, it is a threshold for further partitions, but is based on the minimum loss reduction. Initial search space will be between 0 and 10 with intervals of 0.5.

To introduce randomness, making the training data more robust to noise, the following hyperparameters will also be tuned.

*subsample*—This is the percentage of the training data that is randomly sampled at each boosting iteration. Initial search space will be between 0.5 and 1, with intervals of 0.1.

*colsample_bytree—*Indicates what fraction of columns (features) are selected for tree development per tree. Initial search space will be between 0 and 1, with intervals of 0.1.

*eta*—step size shrinkage. The initial search space will be between 0 and 1, with intervals of 0.1.

Once the restricted search space has been defined, each time the modelling process requires hyperparameter tuning, the grid search will run a total of 729 iterations to find the optimum set of hyperparameters.

All other parameters will be fixed at the default value.

### Development of the model

Conventional modelling strategies involve developing an unadjusted model on the dataset and then evaluating the apparent validity by testing the performance on the same dataset it was developed on. Then, through a process of resampling multiple times, models for each ‘resample’ can be developed by following the exact same modelling steps as in the apparent model. Once this has been completed, the average performance can then act as a penalty on the original model, creating an optimism-adjusted model. This is known as internally validating a model as it has been developed using resampling samples, but from the same data [33]. External validation should occur in a different sample from the development data, and preferably in a different geography and/or time frame [33].

### Apparent validation

In the strategy proposed here, the algorithm does not create an unpenalized model to begin with. This is because tuneable hyperparameters are used to determine *how* the algorithm is developed on the data, prior to model development. In this way, the resultant model is already penalised at the point of development. To obtain the apparent validity of the full model, the three-step process of tuning hyperparameters, building a model on the optimal hyperparameters, and then evaluating the performance will occur on the full dataset. This will be the final model, as it has used the most information of the underlying population in development. The performance however will still be optimistic, even with the tuned hyperparameters as it has been evaluated in the same data it was derived from.

### Internal-external validation

This study benefits from using individual patient data (IPD) from regional datasets clustered by ED. This provides an opportunity for internal-external cross-validation (IECV).

Cross-validation (CV) is a method whereby the data is split into *K* number of partitions (folds) and one-fold is left out as a ‘test set’. The remaining folds are used collectively to train a model. Once the trained model has been applied to the test set, performance measures are recorded, and the set is placed back in the data. The next fold is then held out and the process repeated. This repeats until all folds have been held out. The benefit of cross-validation is that it provides a spread of performance instead of a point estimate. This is useful for indicating model stability.

Nested cross-validation is a variant of CV and consists of an inner-loop and an outer-loop. Like the CV procedure above, the data is split into ten random parts. Then, one tenth is removed as an outer loop test set and the remaining nine tenths are split into random folds again. Due to the quantity of models being developed using this method, this is likely to be 5 random folds. One fifth of the inner loop is removed (inner loop test set), and hyperparameters can be grid-scanned using the data from the remaining four fifths. Optimum hyperparameters are then applied to the inner test set for performance checking. The inner test set is then replaced, and the next fifth removed. The process repeats until all five folds have been removed and tested. The best performing inner loop model has its hyperparameter values applied to the whole inner loop (in-effect, outer-loop training set) to develop a model. This is then applied to the outer-loop test set for model performance. This outer loop tenth is then replaced and the process repeated. Performing nested CV internally validates the model as it is resampling; however, the random splits mean it is not being tested in a new geography.

As a way of simulating this, outer loop test sets are not random but in fact spatial clusters. In this way, the model is being internally-externally validated as it is resampling from the same data but testing it in a new population.

For spatial validation, a different ED will be used for each outer loop holdout. For example, ‘the Sheffield ED model’ will be trained on all EDs except for Sheffield, and then the performance tested on the Sheffield data. There are sixteen EDs in Yorkshire and therefore there will be sixteen spatial clusters.

A limitation of this modelling strategy is the computational expense. For every model, there needs to be 729 models built to identify the optimal hyperparameters. This is then repeated 5 times in each of the inner loops. With 29 clusters (including the full dataset), this means that there will be ~ 102,060 models required to be developed. If it becomes too expensive, then the number of inner loop folds can be reduced from 5 to 3, and any hyperparameter that has the default value as the optimum value in the preliminary search space will become fixed.

The different cluster results will then be pooled into a random-effects meta-analysis [34]. This is to estimate the average performance, the magnitude of heterogeneity between clusters and the range of performance across settings [35]. The predictor effects will not change from the internally validated model, but the performance measures will be updated according to the results of the meta-analysis. It would also be possible to derive a prediction interval for how the model would be expected to perform in a similar population.

### Evaluating the model performance

**Fig. 1:**
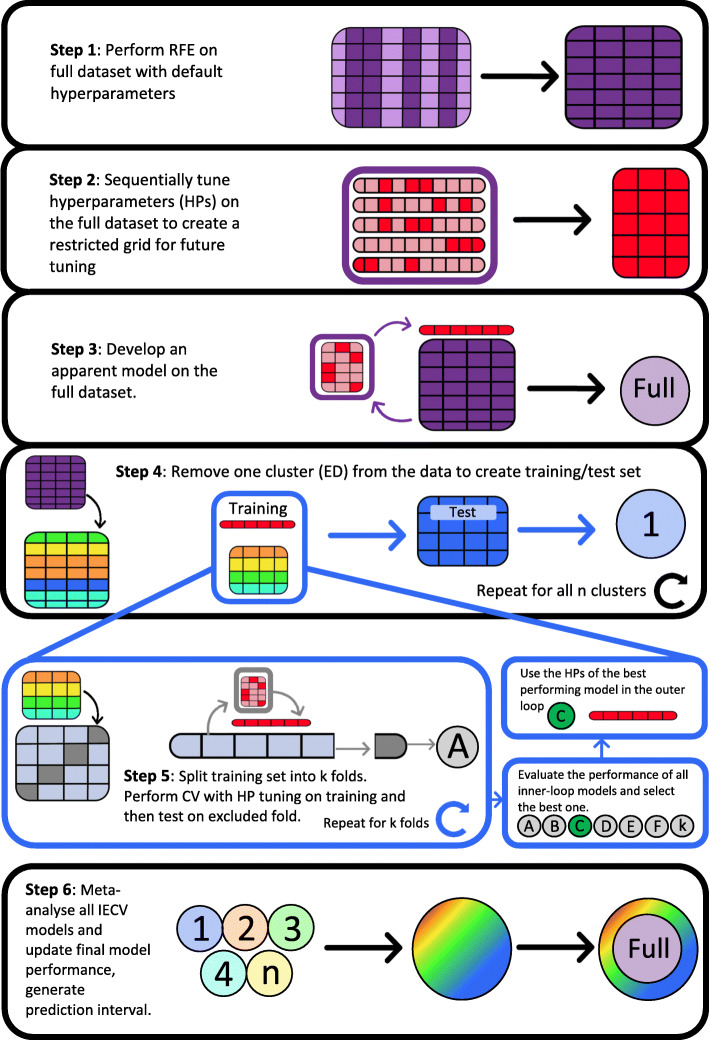
Summary of steps

For hyperparameter tuning, the C-statistic will be used to measure performance. For the apparent and IECV models, there will be three evaluations. The first is the goodness-of-fit as a general measure of model performance. This will be the Cox-Snell pseudo R^2^. For discrimination, the C-statistic will be used and receiver operating characteristic (ROC) curve plotted. For calibration, the plot, intercept and slope will be calculated. All the evaluation metrics will be entered into the meta-analysis to pool and update the performance of the final (full) model. Below is a figure graphically representing the modelling steps (Fig. 1).

## Discussion

### Benefit of a new tool

This study aims to develop a prediction model that can be used to create a tool supporting paramedics in making appropriate and effective decisions for patients who may not require the level of care provided by a hospital. It is important as it is aiming to navigate care decisions that will safely provide patients with the right care, first time. If a paramedic can see the probability that their patient may have an avoidable attendance, it opens an opportunity to explore community options. It also empowers the patient to be an active partner in developing a self-care plan.

It could also have secondary benefits such as freeing ambulance fleet capacity to respond to a patient still waiting for help. With less patients being transported to the ED with low-acuity problems, it could also contribute to minimising delays in care for those who do need specialist ED interventions.

### Presenting the model as a tool

It is anticipated that the prediction model can be presented as a probability of the positive class to the clinician. As an illustrative example, once all predictor variables are inputted into the ePCR by the clinician, it may display the following message—‘The probability of this patient having an avoidable attendance at ED is 32%’.

## Supplementary Information

**Additional file 1.**

## Data Availability

This study uses data from NHS Digital for research purposes and therefore data will not be available following completion of the study in accordance with the data sharing agreement. Findings from the research will be published in a peer-reviewed, open-access journal and disseminated at relevant conferences. The tool will be presented to appropriate stakeholders for real-world prospective evaluation.

## Abbreviations

ECDS: Emergency Care Data Set
ED: Emergency department
CAG: Confidentiality Advisory Group
CDS: Commissioning Data Set
CI: Confidence interval
CV: Cross-validation
ED: Emergency department
EMS: Emergency medical service
ePCR: electronic patient care record
GP: General Practitioner
IECV: Internal-external cross-validation
IPD: Individual patient data
NHS: National Health Service
REC: Research Ethics Committee
ROC: Receiver operating characteristic
TRIPOD: Transparent Reporting of a multivariable prediction model for Individual Prognosis or Diagnosis
YAS: Yorkshire Ambulance Service
