# Supplementary material for: The Safety INdEx of Prehospital On Scene Triage (SINEPOST) study: the development and validation of a risk prediction model to support ambulance clinical transport decisions on-scene—a protocol
Source: Diagn Progn Res. 2021 Nov 8;5:18. doi: 10.1186/s41512-021-00108-4 (PMC8573562; doi:10.1186/s41512-021-00108-4)
Supplement: Supplementary file 1 — Additional file 1. [file 41512_2021_108_MOESM1_ESM.pdf]

Outcome measure and candidate variables

Outcome variable definition

Note that the first attendance and the type of ED are not coded in SNOMED CT and are national codes used in the UK. CS = Code Share and it means the CDS code cannot be used as it is shared with an investigation or treatment that would deem the attendance necessary

Variable	Value	CDS code	ECDS code
Attendance category	Unplanned First Emergency Care Attendance for a new clinical condition (or deterioration of a chronic condition).	1	-
Department type	Type 1: General Emergency Department (24 hour).	1	-
Arrival mode	Arrival by emergency road ambulance	-	1048031000000100
Arrival mode	Arrival by non-emergency road ambulance	-	1048021000000102
Investigation	Clinical investigation not indicated	24	1088291000000101
Investigation	Dementia test	99	165320004
Investigation	Diagnostic dental procedure	22	53115007
Investigation	Glucose measurement, blood, test strip	CS	104686004
Investigation	Human chorionic gonadotropin measurement	21	67900009
Investigation	Peak expiratory flow measurement	99	29893006
Investigation	Urinalysis	6	27171005
Investigation	Urine pregnancy test	21	167252002
Investigation	Urine sent for culture	CS	168338000
Investigation	visual acuity testing	CS	16830007
Investigation	NA	-	-
Treatment	Activities of daily living assessment	521	304492001
Treatment	Application of a dressing, minor	11	15631002
Treatment	Assessment of mobility	91	430481008
Treatment	Closure of skin wound by tape	CS	71810007
Treatment	Dental surgical procedure	56	81733005
Treatment	Gluing of wound	CS	284182000

Treatment	Mobility/transfers education, guidance and counselling	522	410267000
Treatment	New medication commenced	57	266712008
Treatment	Patient given written advice	221	413334001
Treatment	Psychosocial assessment	CS	371585000
Treatment	Review of medication	CS	182836005
Treatment	Social assessment	54	406551008
Treatment	Treatment not indicated	99	183964008
Treatment	Physiotherapy: Falls prevention	92	391027005
Treatment	Observation/ cardiac monitor, pulse oximetry/ head injury / trends	21	88140007
Treatment	NA	-	-
Discharge status	Discharged – follow-up treatment to be provided by general practitioner	2	-
Discharge status	Discharged – did not require any follow-up treatment	3	-
Discharge status	Transferred to other healthcare provider	7	-
Discharge status	Left department before being treated	12	-
Discharge status	Left department having refused treatment	13	-
Discharge status	Left care setting after initial assessment	-	1066311000000101
Discharge status	Left care setting before initial assessment	-	1066301000000103
Discharge status	Left care setting before treatment completed	-	1066321000000107
Discharge status	Streamed from ED to dental service following initial assessment	-	1077051000000105
Discharge status	Streamed from ED to falls service following initial assessment	-	1077091000000102
Discharge status	Streamed from ED to frailty service following initial assessment	-	1077101000000105
Discharge status	Streamed from ED to GP following initial assessment	-	1077021000000100
Discharge status	Streamed from ED to mental health following initial assessment	-	1077041000000107
Discharge status	Streamed from ED to ophthalmology following initial assessment	-	1077061000000108

Discharge status	Streamed from ED to pharmacy service following initial assessment	-	1077071000000101
Discharge status	Streamed from ED to urgent care service following initial assessment	-	1077031000000103
Discharge status	Treatment completed	-	182992009
Discharge Destination	Home	-	306689006
Discharge Destination	Residential care facility without 24 hour nursing care (e.g. residential home)	-	306691003
Discharge Destination	Residential care facility with 24 hour nursing care (e.g. nursing home)	-	306694006
Discharge Destination	Police	-	306705005
Discharge Destination	Custodial services e.g. prison / detention centre	-	50861005
Acuity	Non-urgent	-	1077251000000100

Candidate predictor table with cumulative parameters

Table 2: Demographic variables

Variable category	Variable name (units)	Variable type	Values	Parameters	Cumulative parameters
Demographic	Age (Years)	Continuous	18,19,20 years etc.	1	1
Demographic	Gender	Categorical	Male, Female, Transgender, Unknown	4	5
Demographic	Ethnicity	Categorical	Black, Asian, Mixed, White, Other	5	10
Demographic	Assessment time	Date time	16/07/2019 19:00, 31/01/2020 12:01:00	0	10
Demographic	Weekend flag	Binary	1,0	1	11
Demographic	Out of hours flag	Binary	1,0	1	12
Demographic	Season	Categorical	Summer, Autumn, Winter	4	16
Demographic	Previous attendance within 24 hours	Binary	1,0	1	17
Demographic	Incident location	Categorical	Care home, Domestic address, Not selected, Public place, School, Work, Other	7	24
Demographic	Distance to hospital (Km)	Continuous	1,2,3 Kilometers	1	25
Demographic	Social Deprivation (IMD)	Continuous	1.1, 9.2, 13.4 ...	1	26

Table 3: Social variables

Variable category	Variable name (units)	Variable type	Values	Parameters	Cumulative parameters
Social	GP address recorded	Binary	1,0	1	27
Social	NOK named	Binary	1,0	1	28
Social	Parent named	Binary	1,0	1	29
Social	Guardian named	Binary	1,0	1	30
Social	Social worker named	Binary	1,0	1	31

Table 4: Clinical variables

Variable category	Variable name (units)	Variable type	Values	Parameters	Cumulative parameters
Clinical	Primary survey: Catastrophic haemorrhage	Binary	1,0	1	36
Clinical	Primary survey: Cervical spine tenderness	Binary	1,0	1	37
Clinical	Primary survey: Airway	Categorical	Clear, Noisy, Occluded	3	40
Clinical	Primary survey: Breathing	Categorical	Normal, Abnormal, Not breathing	3	43
Clinical	Primary survey: Pulse	Categorical	Radial, Carotid, No palpable pulse	3	46
Clinical	Primary survey: Level of response	Categorical	Alert, Confusion, Verbal, Pain, Unresponsive	5	51
Clinical	Mental capacity	Binary	1,0	1	52
Clinical	Clinical impression	Categorical	Shortness of breath, Abdominal pain, Hypoglycaemia . . .	97	149
Clinical	Initial pulse rate (bpm)	Continuous	60,61,62 . . .	1	150
Clinical	Initial respiratory rate (rpm)	Continuous	16,17,18 . . .	1	151
Clinical	Initial SpO2 (%)	Continuous	96%,97%,98% . . .	1	152
Clinical	Initial temperature (oC)	Continuous	36.2, 37.1, 37.5 . . .	1	153
Clinical	Initial Systolic BP (mmHg)	Continuous	120,121,122 . . .	1	154
Clinical	Initial diastolic BP (mmHg)	Continuous	80,81,82 . . .	1	155
Clinical	Initial Responsiveness	Categorical	Alert, Confusion, Verbal, Pain, Unresponsive	5	160
Clinical	Initial Glasgow Coma Scale (GCS) score	Categorical	15,14,13,12,11,10,9,8,7,6,5,4,3	13	173
Clinical	Initial GCS: Eye component	Categorical	4,3,2,1	4	177

Clinical	Initial GCS: Verbal component	Categorical	5,4,3,2,1	5	182
Clinical	Initial GCS: Motor component	Categorical	6,5,4,3,2,1	6	188
Clinical	Initial NEWS score	Continuous	1,2,3 . . .	1	189
Clinical	Initial pain score	Continuous	1,2,3..	1	190
Clinical	Hypercapnic respiratory failure	Binary	1,0	1	191
Clinical	Subsequent pulse rate	Continuous	60,61,62 . . .	1	192
Clinical	Subsequent respiratory rate	Continuous	16,17,18 . . .	1	193
Clinical	Subsequent SpO2	Continuous	96%,97%,98% . . .	1	194
Clinical	Subsequent temperature	Continuous	36.2, 37.1, 37.5 . . .	1	195
Clinical	Subsequent Systolic BP	Continuous	120,121,122 . . .	1	196
Clinical	Subsequent diastolic BP	Continuous	80,81,82 . . .	1	197
Clinical	Subsequent Responsiveness	Categorical	Alert, Confusion, Verbal, Pain, Unresponsive	5	202
Clinical	Subsequent Glasgow Coma Scale (GCS) score	Categorical	15,14,13,12,11,10,9,8,7,6,5,4,3	13	215
Clinical	Subsequent GCS: Eye component	Categorical	4,3,2,1	4	219
Clinical	Subsequent GCS: Verbal component	Categorical	5,4,3,2,1	5	224
Clinical	Subsequent GCS: Motor component	Categorical	6,5,4,3,2,1	6	230
Clinical	Subsequent NEWS score	Continuous	-1,0,1 . . .	1	231
Clinical	Subsequent pain score	Continuous	1,2,3..	1	232
Clinical	Difference pulse rate	Continuous	-1,0,1 . . .	1	233
Clinical	Difference respiratory rate	Continuous	-1,0,1 . . .	1	234
Clinical	Difference SpO2	Continuous	-1,0,1 . . .	1	235
Clinical	Difference temperature	Continuous	-1,0,1 . . .	1	236

Clinical	Difference Systolic BP	Continuous	-1,0,1 . . .	1	237
Clinical	Difference diastolic BP	Continuous	-1,0,1 . . .	1	238
Clinical	Difference Responsiveness	Continuous	-1,0,1 . . .	1	239
Clinical	Difference Glasgow Coma Scale (GCS) score	Continuous	-1,0,1 . . .	1	240
Clinical	Difference NEWS score	Continuous	-1,0,1 . . .	1	241
Clinical	Difference pain score	Continuous	1,2,3..	1	242
Clinical	Initial peak flow	Continuous	300,301,302 . . .	1	243
Clinical	Subsequent peak flow	Continuous	300,301,302 . . .	1	244
Clinical	Difference peak flow	Continuous	300,301,302 . . .	1	245
Clinical	Initial pupil reaction left	Binary	1,0	1	246
Clinical	initial pupl reaction right	Binary	1,0	1	247
Clinical	subsequent pupil reaction left	Binary	1,0	1	248
Clinical	subsequent pupil reaction right	Binary	1,0	1	249
Clinical	difference pupil reaction left	Binary	1,0	1	250
Clinical	difference pupil reaction right	Binary	1,0	1	251
Clinical	initial pupil size left	Continuous	1,2,3 . . .	1	252
Clinical	initial pupil size right	Continuous	1,2,3 . . .	1	253
Clinical	subsequent pupil size left	Continuous	1,2,3 . . .	1	254
Clinical	subsequent pupil size right	Continuous	1,2,3 . . .	1	255
Clinical	difference pupil size left	Continuous	1,2,3 . . .	1	256
Clinical	difference pupil size right	Continuous	1,2,3 . . .	1	257
Clinical	Referral to service	Categorical	Coroner, Police, Safeguarding adult, Safeguarding child . . .	5	262
Clinical	Abnormal ECG on primary	Categorical	Left Bundle Branch Block, Right BBB, STEMI	3	265

Table 5: Interventional variables

Variable category	Variable name (units)	Variable type	Values	Parameters	Cumulative parameters
Interventional	ECG monitored	Binary	1,0	1	266
Interventional	Supplemental oxygen	Binary	1,0	1	267
Interventional	ICN type	Categorical	Intravenous, Intraosseous, None	3	270
Interventional	Drug 1	Categorical	Adrenaline 1:1000, Co-codamol 30/500, Diazepam …	100	370
Interventional	Drug 2	Categorical	Adrenaline 1:1000, Co-codamol 30/500, Diazepam …	100	470
Interventional	Drug 3	Categorical	Adrenaline 1:1000, Co-codamol 30/500, Diazepam …	40	510
Interventional	Drug 4	Categorical	Adrenaline 1:1000, Co-codamol 30/500, Diazepam …	25	535
Interventional	Drug 5	Categorical	Adrenaline 1:1000, Co-codamol 30/500, Diazepam …	17	552
Interventional	Drug 6	Categorical	Adrenaline 1:1000, Co-codamol 30/500, Diazepam …	16	568
Interventional	Drug 7	Categorical	Adrenaline 1:1000, Co-codamol 30/500, Diazepam …	14	582
Interventional	Drug 8	Categorical	Adrenaline 1:1000, Co-codamol 30/500, Diazepam …	12	594
Interventional	Airway type	Categorical	ETT, LMA, OPA, NPA…	10	604

Interventional	Immobilisation	Categorical	Scoop, Cervical collar, Extrication board . . .	9	613
Interventional	Advice given	Categorical	Wound care, bereavement, head injury	8	621
Interventional	Mobility	Categorical	Stretcher, walked, hoist	15	636
Interventional	CPR	Binary	1,0	1	637
